# Avibase – a database system for managing and organizing taxonomic concepts

**DOI:** 10.3897/zookeys.420.7089

**Published:** 2014-06-25

**Authors:** Denis Lepage, Gaurav Vaidya, Robert Guralnick

**Affiliations:** 1 Bird Studies Canada, P.O. Box 160, 115 Front St., Port Rowan, ON Canada N0E 1M0; 2 Department of Ecology and Evolutionary Biology, and CU Museum of Natural History, University of Colorado Boulder, Campus Box 265, Boulder, CO, United States of America 80309-0265

**Keywords:** Biodiversity informatics, scientific names, taxon circumscription, taxonomic concepts, taxonomic database

## Abstract

Scientific names of biological entities offer an imperfect resolution of the concepts that they are intended to represent. Often they are labels applied to entities ranging from entire populations to individual specimens representing those populations, even though such names only unambiguously identify the type specimen to which they were originally attached. Thus the real-life referents of names are constantly changing as biological circumscriptions are redefined and thereby alter the sets of individuals bearing those names. This problem is compounded by other characteristics of names that make them ambiguous identifiers of biological concepts, including emendations, homonymy and synonymy. Taxonomic concepts have been proposed as a way to address issues related to scientific names, but they have yet to receive broad recognition or implementation. Some efforts have been made towards building systems that address these issues by cataloguing and organizing taxonomic concepts, but most are still in conceptual or proof-of-concept stage. We present the on-line database Avibase as one possible approach to organizing taxonomic concepts. Avibase has been successfully used to describe and organize 844,000 species-level and 705,000 subspecies-level taxonomic concepts across every major bird taxonomic checklist of the last 125 years. The use of taxonomic concepts in place of scientific names, coupled with efficient resolution services, is a major step toward addressing some of the main deficiencies in the current practices of scientific name dissemination and use.

## Introduction

The ability to unambiguously describe a concept through nomenclature is fundamental to science. This is particularly true for organizing information about global biodiversity (Patterson et al. 2010); yet, scientific names of biological organisms often poorly resolve the concepts they are intended to describe (Kennedy et al. 2006, Franz and Thau 2010, Franz and Cardona-Duque 2013). In a review of 12 successive classifications of German mosses published over 73 years, Geoffroy and Berendsohn (2003) found that a mere 13% of 1548 taxonomic entities remained consistent in both name and circumscription. In a comparison of North American vascular plant taxonomies published between 1927 and 2006, Franz et al. (2008) found that only 55% of taxa remained unchanged. As the use of database systems for managing vast amounts of biodiversity data becomes increasingly prevalent, there is a strong need for a system designed to organize the millions of taxonomic entities with which the diversity of life is catalogued, and for resolving the meanings behind their names.

A fundamental problem with taxonomic names is that they only refer unambiguously to type specimens, instead of the biological circumscriptions that underlie most name usages. Because our views of these circumscriptions are constantly being challenged and redefined, the circumscriptions attached to a valid name may change dramatically without any change in the name itself. This issue is of great practical importance to people building large-scale biodiversity repositories. Key biological features, such as geographic ranges or overall genetic variability, are shared properties of taxon circumscriptions, not names. As more aggregate trait and phylogenetic databases are published, it is essential to ensure that producers and consumers have clear ways to understand the circumscriptions being used. For managers of biological collections databases, the fluid definitions of names are furthermore compounded by issues such as homotypic and heterotypic synonymy, homonymy and emendations of names based on the rules of the codes of nomenclature. There are millions of valid species and subspecies names and probably an even greater number of proposed names that have later been placed in synonymy, in addition to the many orthographic variants because of a change in genus, changes in gender agreement or other emendations (e.g. David and Gosselin 2002).

Carefully constructed nomenclatural databases with resolution services for homonyms and synonyms, such as the one proposed by the Global Names Architecture (Patterson et al. 2010), can go a long way to addressing these issues. Equally important will be efforts to semantically model the processes and results of taxonomic effort, leading to ontologies for taxonomic names (Franz and Peet 2009, Franz and Thau 2010) and tools such as the Euler/ASP toolkit (Chen et al. 2014, Franz et al. 2014). Such ontologies help ensure interoperability across individual implementations, a much-needed step given the scope of the problem of aggregating taxon names. Fundamental to the development of these projects is the notion of taxonomic concepts (Berendsohn 1995, Kennedy et al. 2006), which have been proposed as a solution to the issue of the ever-changing usage of names. They refer to a scientific name’s underlying circumscription by providing a reference to an author and publication where this circumscription is defined, or from which it can be inferred. For instance, the name *Parus major* Linnaeus, 1758 *sec.* Clements 2000 refers to the circumscription of the Great Tit as inferred from the Clements Checklist of the Birds of the World, 5^th^ edition (Clements 2000). This taxonomic concept can be said to be congruent with *Parus major* Linnaeus, 1758 *sec.* Dickinson 2003 as both refer to congruent sets of individuals, but only partly overlapping with *Parus major* Linnaeus, 1758 *sec.* Gill and Donsker 2013, which has a much more restricted range and is entirely included in the former.

### The limitations of taxonomic concepts

Although taxonomic concepts address some of the limitations of names, they have limitations and issues of their own. While taxonomic concepts have the theoretical advantage of removing the ambiguity associated with scientific names, they are most useful when the relationships between overlapping concepts are well understood. A significant challenge is that there are potentially many more taxonomic concepts than there are taxonomic names, requiring descriptions of relationships between overlapping concepts. Most development regarding taxonomic concepts has focused on establishing relationships between pairs of concepts, whether within a particular publication (vertical relationships) or among different taxonomic publications (horizontal relationships). The relationships between concepts can be expressed with predicates that describe the degree of congruency (Franz and Peet 2009). While this provides an invaluable framework and is a necessary step to developing more detailed formal ontologies, it can be very difficult to scale up given that the number of pairwise comparisons grows at an exponential rate and there are potentially hundreds of sources of taxonomic concepts and thousands of concepts to compare.

Birds provide an excellent example of the challenges inherent in managing taxonomic concepts. There are at least eight major global and widely used taxonomic authorities that have published checklists of taxonomic names (and thus concepts) encompassing all known bird species, several regional authorities that have focused exclusively on a particular geographic area (e.g. Christidis and Boles 2008), and countless more specialized publications that have focused on specific taxonomic groups or individual species. Most sources have published several major versions of the same checklist over the course of several decades, as well as many other minor revisions. The American Ornithologists’ Union’s Checklist of North American Birds, for example, has published seven full checklist editions and 54 partial revisions between 1886 and 2013.

Birds stand out from many other taxonomic groups because they are well studied, and multiple taxonomies curated by multiple sources are available. This also creates a large number of concepts to organize for any given name. Concept management for such a well-studied taxonomic group is particularly challenging because a simple solution, such as documenting only taxonomic changes instead of recording every concept in every checklist, is not feasible for several reasons. First, such a solution assumes that there is a strictly linear temporal sequence in publications, something that only applies within a given authority, such as Clements or the American Ornithologists’ Union, and does not help resolve relationships between independent authorities or even pairs of non-consecutive publications within the same authority. Secondly, because concepts from taxonomic publications may be used as proxies to refer to original concepts, all taxonomic concepts from a publication at a particular point in time need to be resolvable, not only those that are representing changes from earlier publications. While this information is rarely provided in published taxonomic data, data custodians will generally be able to identify which authority and version they were using to describe names at the time their data were curated.

## Avibase, the World Bird Database

Avibase (Lepage 2014) is a large taxonomic database system that attempts to organize all authoritative avian taxonomic concepts, particularly those published in the form of comprehensive global or regional checklists (relational concepts, *sensu* Franz and Peet 2009). Avibase contains taxonomic concepts from 151 taxonomic checklists published in the last 125 years by 17 different authorities (Table 1), as well as taxonomic concepts from other sources. These cover both global checklists (e.g. Howard and Moore, Clements, and the International Ornithological Committee checklists) and regional checklists (e.g. the British Ornithologists’ Union and the American Ornithologists’ Union’s North and South American Classification Committees) of all birds known and currently recognized, including both the original publication of these checklists and all subsequent revisions. These currently represent over 844,000 taxonomic concepts for species and 705,000 concepts for subspecies (there are typically about twice as many recognized subspecies of birds as there are species, but not all authorities include subspecies in their treatment).

**Table 1.** Source of taxonomic concepts included in Avibase, with the number of versions published (including major editions as well as minor revisions). * indicates regional checklists only covering species for a specific part of the world.

Checklist source	Publ. Years	N of versions (incl. revisions)
African Bird Club *	2004–2010	6
American Ornithologists' Union *	1886–2013	61
Birdlife	2007–2012	6
British Ornithologists' Union *	2006–2009	2
Christidis and Boles (Australia) *	2008	1
Commission internationale pour les noms français des oiseaux	1993–2009	2
Clements Checklist of Birds of the World	1974–2013	18
eBird Checklist	2010–2013	4
Howard and Moore	1980–2008	11
Handbook of the Birds of the World	1992–2011	1
International Ornithological Committee	2006–2012	22
Morony, Bock and Farrand	1975	1
Oriental Bird Club *	2001	1
James Lee Peters	1931–1987	1
Sibley and Monroe	1993–1998	3
South American Classification Committee *	2003–2013	11
Zoonomen – Zoological Nomenclature Resource	2007	1

**Figure 1.**
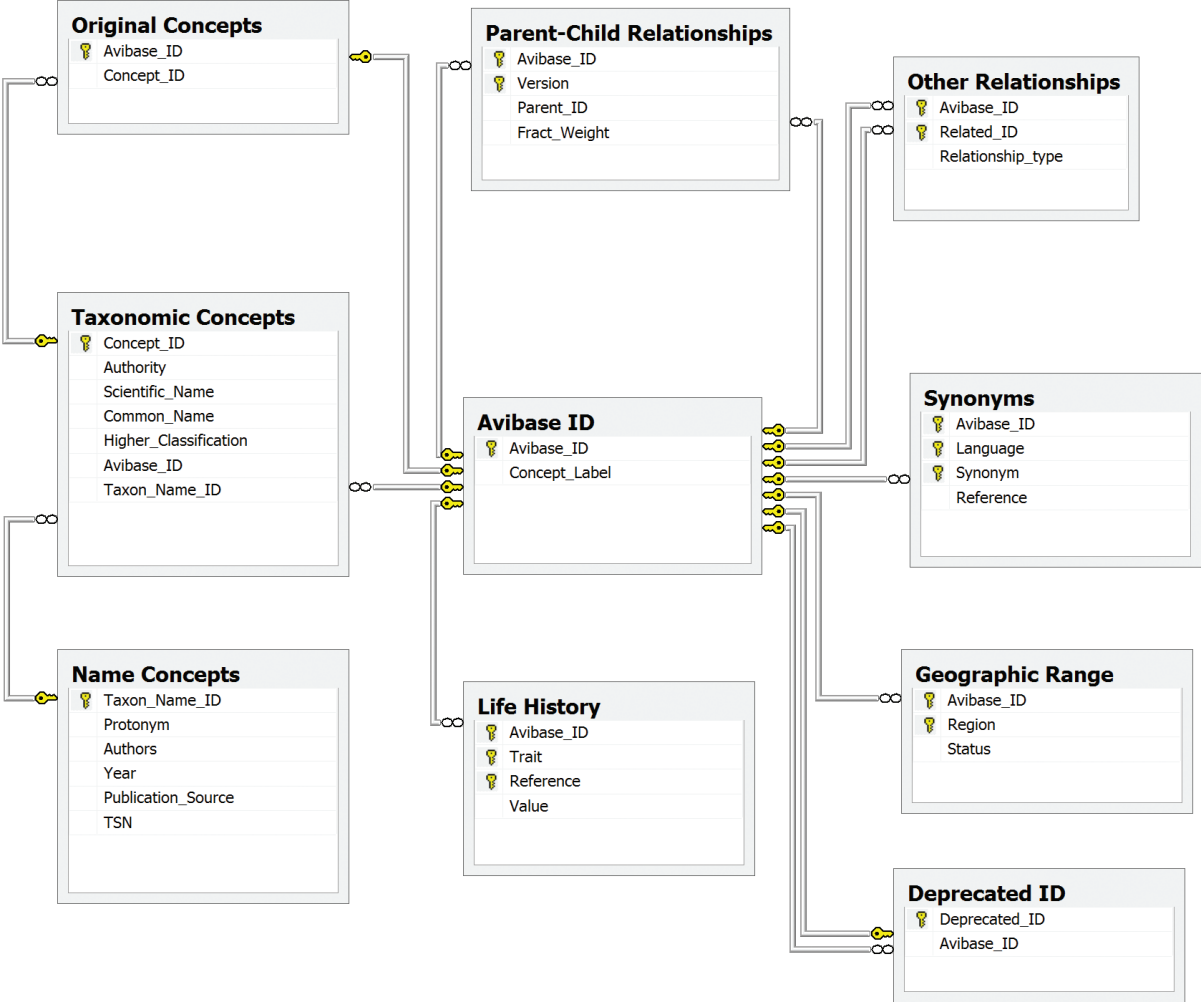
Simplified schema of Avibase primary tables, The Avibase ID table is the central element of Avibase, to which all other concepts are related, and which aims to represent all distinct taxonomic concepts ever published for birds. Published **taxonomic concepts** (species and subspecies, as well as subspecies groups in some cases), along with their scientific and common names as recognized in the publication, are each mapped to a single Avibase ID. A table of **parent-child relationships** is used to describe relationships between different Avibase IDs. Because all taxonomic concepts are congruent with Avibase IDs, relationships among taxonomic concepts themselves are not needed. Biological properties (geographic range, life-history, etc.) are linked directly to Avibase ID, as are **synonyms**, a table that partly overlaps with the names used by taxonomic concepts, but that can also extend to vernacular names in multiple languages. **Name concepts**, which relate to names attached to original type specimens, are a property of taxonomic concepts, and can themselves be linked to ITIS Taxonomic Serial Number (TSN) identifiers.

At the heart of Avibase is the notion of transparent and consistent representation of distinct taxonomic concepts. While there are vast numbers of taxonomic concepts in the "shallow" sense of unique name/source combinations (concept labels), there are far fewer "deeper", taxonomically unique (non-congruent) concept clusters that represent unique circumscriptions. Avibase assigns a unique database identifier to each of these distinct concept clusters (called an Avibase ID), composed of a random hexadecimal key (e.g. 2624054ED644AABB). The table of Avibase IDs, the central component around which the entire database is constructed (Fig. 1), attempts to capture all distinct taxonomic concepts ever published in those major authoritative sources. If one includes all taxonomic concepts that have been originally published as species and subspecies, as well as superspecies, subspecies groups, hybrid forms and phenotypic forms (sometimes originally described as valid species), there are 50,696 unique taxonomic concepts that have so far received an Avibase ID. Of these, 38,755 are from the 151 bird checklists in Avibase; the remaining 11,941 concepts are from other publications or represent unique taxon assemblages and were added separately (Table 2).

**Table 2.** A breakdown of distinct taxonomic concepts in Avibase. 38,755 distinct concepts were obtained from the 151 bird checklists listed in Table 1; the remaining 11,941 were described elsewhere (e.g. hybrids) or represent unique taxon assemblages (e.g. groups of species) and were added to the database separately. Note that although a distinct Avibase ID denotes a congruent circumscription cluster, it need not indicate the same rank: for example, 1,634 concepts were described by some authorities as species and by others as subspecies, while still denoting congruent sets of individuals. Other taxonomic treatments within checklists refer primarily to subspecies and species groups, as well as to distinct phenotypic forms.

Treatment in checklists	Number of concepts
Species only	10,964
Subspecies only	22,477
Other only	1,474
Species or subspecies	1,634
Species or other	468
Subspecies or other	961
Species, subspecies or other	777
Only Avibase IDs	
New taxa, formally described	3
New taxa, not yet formally described	33
Doubtful or invalid taxon	93
Genera (including extinct and synonyms)	3,934
Species groups (e.g. superspecies)	252
Subspecies groups	265
Species hybrids	3,231
Subspecies intergrades	55
Subspecies (junior synonyms)	3,014
Fossil species or subspecies	1,027
Phenotypic forms	34
Subtotal (only Avibase IDs)	11,941
Total	50,696

Some distinct taxonomic concepts share the same scientific name across all checklists: for example, the name *Nycticorax nycticorax* refers to a congruent circumscription cluster and maps to the same Avibase ID in all 151 checklists within Avibase. However, such concordance among authorities is far from the norm: only 11 of the 19,260 unique combinations of circumscriptions and scientific names for species (bearing both the same exact name and the same Avibase ID) have been used by all 151 authorities. Many circumscriptions can therefore bear several distinct names (concept synonyms), and the same names can often be used to describe different circumscriptions (concept homonyms). For instance, the names *Francolinus gariepensis*, *Francolinus levaillantoides*, *Francolinus levaillantoides*, *Scleroptila gutturalis* and *Scleroptila levaillantoides* can all refer to a congruent circumscription cluster (Avibase ID 8E833C63E70A547C), whereas the name *Puffinus lherminieri* can refer to up to 12 distinct circumscriptions. When restricting this analysis to the 70 global authorities, we found 18,278 unique combinations of scientific species names and distinct taxonomic concepts, with 4,451 being used in all 70 cases, less than half of the 10,000 currently recognized species.

For managers of biological data collections, the benefits of having a permanent identifier for a stable biological unit that is not subject to the changing nature of names or circumscriptions are apparent. Databases which only track names and their synonyms, such as the Integrated Taxonomic Information System (ITIS), cannot address the ambiguities of circumscriptions. In Avibase, a unique Avibase ID always denotes a distinct, unique circumscription cluster. Congruent taxonomic concepts by definition share all their biological properties because they refer to taxonomically congruent sets of individuals. Ecological and biological traits such as geographic distribution, life-history characteristics, genotypes, behavior, and ecological preferences are all properties of the biological circumscription, linked together in Avibase by the Avibase ID, instead of being attached to names that may change in either orthography or definition (Fig. 1).

### Concept trees

Because each Avibase ID identifies a distinct taxonomic concept, and many published taxonomic concepts are congruent to each other, it is sufficient to build entity relationships among Avibase IDs rather than among all taxonomic concepts individually. This greatly reduces the number of relationships to describe. The primary type of relationship that has been implemented in Avibase is the direct one-to-many parent-child relationship between Avibase IDs, corresponding to the “Includes” (>) predicate *sensu* Franz and Peet (2009), with the main exception of hybrids. When built in hierarchical trees, which we refer to as “concept trees”, direct parent-child relationships allow programmatic derivation of many of the other types of relationships, such as indirect descendants, partial overlap between concepts, congruency, and exclusion. We discuss this in more detail below.

**Figure 2.**
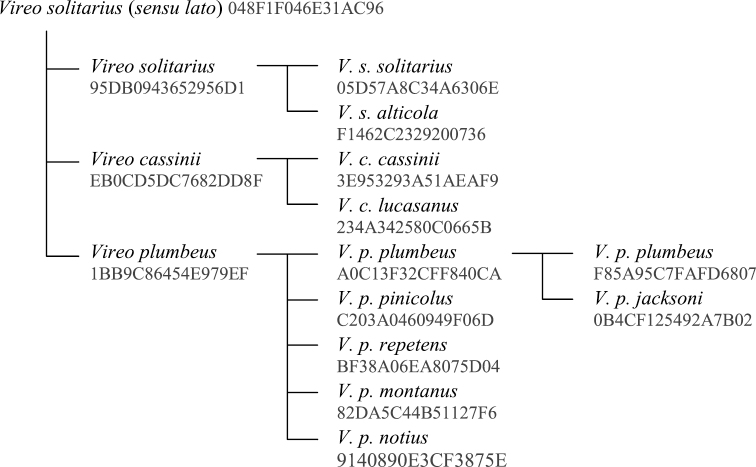
The relationships between the taxonomic entities related to the *Vireo solitarius* superspecies. Numbers under each name refer to Avibase IDs. The concept for the nominal *plumbeus* *sec.* Oberholser, 1974 is therefore distinct from the concept for the nominal subspecies *plumbeus* when *jacksoni* is subsumed. It is worth noting however that the recognition or not of *jacksoni* does not affect the higher related concepts, such as the *plumbeus* species, any of its other subspecies, or the rest of the *Vireo solitarius* complex because they are either orthogonal to those alternative arrangements or they completely include both. In the database model (Fig. 1), these trees are maintained with the parent-child relationship table in which each Avibase ID only needs to identify its immediate parent, while other relationships can be calculated programmatically.

As an example of a concept tree, we consider the Solitary Vireo complex. The 6^th^ edition of the AOU North American Checklist (American Ornithologists’ Union 1983) recognized a single species in this complex, *Vireo solitarius*. In the Forty-first Supplement (Banks et al. 1997), two groups of subspecies within this species were raised to full species: *Vireo plumbeus* (including subspecies *plumbeus*, *pinicolus*, *repetens*, *montanus* and *notius*) and *Vireo cassinii* (including subspecies *cassinii* and *lucasanus*). There are therefore two separate taxonomic concepts in Avibase, with separate Avibase IDs but the same name *Vireo solitarius*: one represents *Vireo solitarius* *sensu lato*, which includes *plumbeus* and *cassinii*, and the other represents *Vireo solitarius* *sensu stricto*, which does not overlap with *plumbeus* and *cassinii*. In Avibase, all three forms of the Solitary Vireo (*Vireo plumbeus*, *Vireo cassinii* and *Vireo solitarius* *s.s.*) are children of *Vireo solitarius* *s.l.* (Fig. 2). Each of these three forms can in turn have their own subspecies. This type of relatively simple model with a single tree represents a majority of relationships among related taxonomic concepts.

Avibase IDs are only needed for the smallest operational unit used by concept publishers, which in the case of birds is usually the species or the subspecies, and relationship trees will generally only need to capture relationships among taxa within a superspecies group or among species that have been historically considered as conspecific. Avibase IDs can also be created for other taxonomic levels, such as genera, or even for arbitrary taxonomic arrangements such as subspecies groups or pairs of species that might be confused in the field but are not necessarily genetically close, as is used extensively in the eBird taxonomy (Clements et al. 2013). A disadvantage of creating Avibase IDs for higher taxonomic levels that comprise many children (e.g. family) is that they tend to be challenging to manage, because of the higher number of possible combinations of children at those levels, with each combination representing a possible circumscription requiring an Avibase ID. Fortunately, some of the tasks that one may want to perform with those higher taxonomic levels can also be achieved more simply without creating Avibase IDs. For example, for evaluating whether genus or family concepts are congruent between distinct authorities, one could programmatically look at whether they are comprised of the same trees or portions of trees in each authority.

### Alternate concept trees

**Figure 3.**
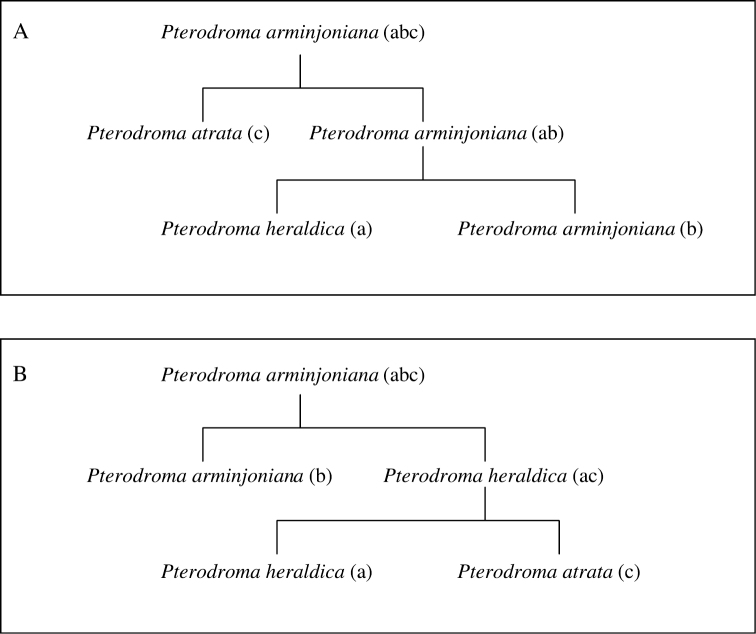
The relationships between *Pterodroma arminjoniana*, *Pterodroma heraldica* and *Pterodroma atrata*, with two alternative arrangements (**A** and **B**) of biological concepts found in taxonomic authorities. Concepts with the same lowercase letters in brackets in the two diagrams represent congruent circumscriptions.

Alternate concept trees are required in those relatively rare cases (5.7% of all Avibase IDs) where several mutually contradictory arrangements have been proposed. For instance, the superspecies *Pterodroma arminjoniana* is now considered three distinct species (*Pterodroma arminjoniana*, *Pterodroma heraldica* and *Pterodroma atrata*), but several arrangements of those have been proposed which involve at least two different relationship trees (Fig. 3): one in which *atrata* is included in *heraldica* *s.l.* and one in which it is not. From these two alternative taxonomic trees, four different valid combinations of taxonomic concepts are possible and have been published within the same checklist: 1) the superspecies *Pterodroma arminjoniana* (abc) alone, 2) *Pterodroma arminjoniana* (ab, including *heraldica*) and *Pterodroma atrata* (c), 3) *Pterodroma heraldica* (ac, including *atrata*) and *Pterodroma arminjoniana* (b) and 4) *Pterodroma heraldica* (a), *Pterodroma arminjoniana* (b) and *Pterodroma atrata* (c) as distinct species. The use of those concepts by various checklists can be visualized in a grid (http://avibase.bsc-eoc.org/species.jsp?avibaseid=A26C9D6B5C859E5E&sec=taxontable). Note that in all four combinations of concepts, the three letters representing the finer levels (a, b and c) are always included, and included only once. This property of the relationship trees can be used to validate the arrangements and map the taxonomic concepts to Avibase IDs (see section “*Validating parent-child relationships across checklists using fractional weights*”). One should also note that taxon concept combinations 1 and 4 are present in both taxonomic trees A and B. In checklists that use those combinations, it is not possible to determine which alternate tree applies, nor is it necessary because both trees lead to the same solution for mapping taxonomic concepts and Avibase IDs.

Alternate concept trees are not necessary in all cases of taxonomic revision. For instance, if two populations previously treated as part of the same subspecies are split into two subspecies, it suffices to extend the terminal branch of the tree for that species with new child nodes, without affecting any of the higher levels or the rest of the tree structure, as all previously existing nodes continue to refer to congruent concept sets. Likewise, independent trees that do not share nodes can be merged together to form a larger tree (e.g. if two species were found to have close affinity and lumped together). It is also possible to create new nodes within the tree by grouping existing branches together (such as by lumping *Vireo cassinii* and *Vireo plumbeus* together, but without *Vireo solitarius* in Fig. 2). However, any taxonomic changes that require moving nodes to different branches, such as reassigning a subspecies to a different species, will require a new tree, and most likely will also require new nodes to represent these new combinations. Generally speaking, a model that minimizes the number of alternate trees is greatly preferable, whenever this can be achieved.

In Avibase, alternate trees sharing some of the same branches or nodes are constructed in such a way that they also share the same root taxon node. That is, each taxon should have the same highest-level root parent concept in all alternate trees. In some cases, this could mean building trees representing large species complexes that are frequently lumped together in various arrangements – in birds, the species complexes for *Larus argentatus* *s.l.*, *Puffinus lherminieri* *s.l.*, and *Otus magicus* *s.l.* are some of the most elaborate examples with up to a dozen possible candidate species, many subspecies, nine node levels and up to five possible alternate relationship trees. This may require creating Avibase IDs for concepts that have never been suggested historically as representing a distinct taxon on their own, and for which a new labeling system may be required (Franz et al. 2014). For instance, one can imagine two genetically related species A and B (e.g., part of the same genus) that have never been considered as part of a superspecies or lumped under the same species, and therefore being top nodes of unrelated trees each containing several subspecies. If, following a scientific study, a checklist suggests moving some of the subspecies of species B under species A, new species-level concepts A’ (containing the original subspecies of A plus the ones moved from B) and B’ (containing the original subspecies of B minus those moved to A) will need to be created, each with a new combination of subspecies. Rather than maintaining 4 distinct trees (with top nodes A, B, A’ and B’), a more useful approach is to create a new parent concept that encompasses the entire group (A+B), and that can serve as the top node for a tree that includes both A and B, and another tree that includes concepts A’ and B’, as well as their respective subspecies. Doing so allows easy identification of related concepts because they share the same top root node, which can be invaluable when trying to define the types of relationships described in Franz and Peet (2009), such as partial overlaps, additions, and subtractions. This approach is also required for the use of fractional weights as a mean of validating and facilitating concept mapping and relationships, which is described in more detail below.

## Mapping taxonomic concepts in Avibase

As new taxonomic checklists are published, each taxonomic concept they contain must either be mapped to an existing Avibase ID or have a new Avibase ID assigned to it. Avibase treats each partial revision of a checklist as if it was published in full again but with the changes implemented. For instance, when the AOU publishes a limited list of annual revisions, it implicitly leaves all other concepts unchanged. While it may seem redundant to repeat all taxonomic concepts at each revision, including the ones that did not change, this process greatly simplifies identifying concepts in use at a given point in time. For authorities that do not have a versioning approach and where corrections are gradually implemented as they are acted upon throughout the year, such as the South American Classification Committee, Avibase periodically freezes a version arbitrarily (in this case, about once a year). We strongly encourage publishers of taxonomic concepts to apply a consistent versioning approach and to maintain and make available archived versions.

In all cases, the process of mapping concepts is most easily done by comparing a new checklist with another one already mapped in Avibase, preferably one that uses similar taxonomic treatments. If the new checklist is a relatively minor revision of an existing checklist already mapped in Avibase, most of its taxonomic concepts will have the same scientific or common name, a congruent biological meaning and will map to the same Avibase IDs, thereby greatly simplifying the work.

The database manager handling the addition would initially attempt to match all scientific names of all concepts (species and subspecies) shared by the two checklists, and look for differences. Changes in scientific name alone, such as reassignment to a new genus or a change in the spelling of the epithet to reflect gender agreement, do not warrant a change in Avibase ID but do complicate the initial matching of the two checklists. This issue can be addressed by manual inspection or by relying on common names, other identifiers provided by the publisher, or a table of scientific name synonyms. Creating a new Avibase ID is required only where the biological underpinning of a name has changed, such as following additions to the checklist (e.g. new species) or taxonomic splits, lumps, and partly overlapping relationships, which can often be easily detected by looking for any additions or deletions of concepts or reassignments of subspecies. In many cases where a new Avibase ID is required, a congruent taxonomic concept will already have been defined by another authority and mapped to an existing Avibase ID: for example, in the 2013 version of the Clements checklist (Clements et al. 2013), there were 10,324 species listed, including 176 (1.7%) that were not in the previous edition (Clements et al. 2012). Since other checklists already contained these concepts, only 41 (23.3%) represented concepts entirely new to Avibase for which new Avibase IDs were needed.

Concept publishers, in their justification for taxonomic changes, often provide the information necessary to identify the circumscription intended by a taxonomic concept. For instance, they may explicitly say that they are splitting or lumping concepts to create new ones. Other information, such as phenotypic descriptions (plumage, song, behavior, etc.) and geographic range, can also be used to assess whether taxonomic concepts are congruent with those of other authorities. The examination of other concepts within the same checklist can also reveal implicit circumscriptions: for example, *Parus major* *s.l.* sometimes contains *Parus major* *s.s.* and *Parus cinereus*. A checklist that contains *Parus major* but not *Parus cinereus* is probably referring to *Parus major* *s.l.* and not *Parus major* *s.s.* The list of subspecies assigned to a species may be useful in identifying its circumscription. Ultimately, this process in Avibase requires some level of manual processing on the part of a database manager with some knowledge of the taxonomic group. While properties of concepts and concept trees can help identify and validate candidate concepts for mapping, expert knowledge is necessary for proper curation.

### Validating parent-child relationships across checklists using fractional weights

**Figure 4.**
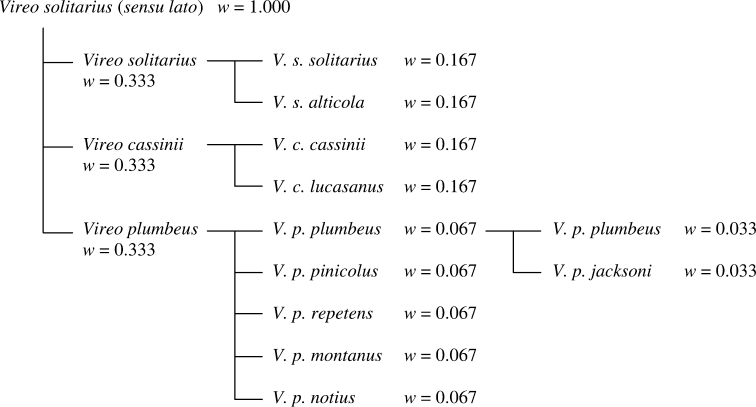
Fractional weights (w) can be used to validate the taxonomic arrangements within a particular authority. In this example, in any valid listing of the concepts within an authority, the sum of all taxonomic concepts related to the superspecies *Vireo solitarius* should add up to 1.0 at both the species and the subspecies levels.

Parent-child relationships and concept trees may be used both to identify relationships in new publications and to validate existing relationships. The algorithm used by Avibase assigns and stores fractional weights for each node of a concept tree, starting with a weight of 1.0 for the top concept in a tree. Child nodes recursively receive an equal fraction of the weight of their parent. *Vireo solitarius*, *cassinii* and *plumbeus*, for instance, would each receive a weight of 0.333 (Fig. 4). The two subspecies of *solitarius* and *cassinii* would each receive a weight of 0.167 (half of their parent node), and the five recognized subspecies of *plumbeus* would each receive (0.067) (one-fifth of their parent node). Finally, the subsumed subspecies *jacksoni* and the nominal *plumbeus* that excludes *jacksoni* would each receive a weight of 0.033. Within any authority with global coverage, such as a global bird checklist, there should always be at least one alternate tree for which the sum of weights yields a total of 1.0 for a suite of related species concepts. In the *Vireo* example, the two valid options at the species level are listing *Vireo solitarius* *s.l.* alone (total weight = 1.0), or the three forms individually (total weight = 0.333 + 0.333 + 0.333 = 1.0). For authorities that are restricted in coverage or incomplete in scope, such as a checklist of North American birds, the same approach can be used but with weights recalculated to exclude portions of the trees that are not covered by the scope of the authority. It is possible for a set of incorrect arrangements to add up to 1.0 by chance; Avibase uses a series of rules (listed in Table 3) to detect such cases.

**Table 3.** To be valid, an assemblage of related taxonomic concepts within an authority should follow at least the following set of rules, which emerge as logical constructs from the database design. While these rules are not strictly enforced in Avibase, any deviations would suggest either a problem with the mapping of taxonomic concepts to Avibase IDs, or a problem in the concept trees of Avibase IDs.

1.	The sum of fractional weights for species-level taxonomic concepts mapping to Avibase IDs within the same tree should be equal to 1.0 in at least one particular complete taxonomic arrangement and should never exceed 1.0, even in alternative trees. This rule should also hold above the species level, but as higher taxa can generally be completely specified by listing the species within them, such an approach is probably unnecessary.
2.	In cases where there are alternative trees, only a single mapping of taxonomic concepts to Avibase IDs should provide a sum of 1.0 on one or more of the alternate trees. In the example of *Pterodroma* (Fig. 3), concepts a, b and c are present and validly arranged in both trees, and both will offer the same solution in term of mapping taxonomic concepts to Avibase IDs when all three species are present.
3.	The sum of weights for subspecies concepts in a given checklist should equal the weight of the species in which they are included. In other words, if a checklist includes a concept for *Vireo plumbeus* that maps to an Avibase ID with a weight of 0.33 in a given tree, the sum of the Avibase ID weights for the subspecies of *Vireo plumbeus* in the same checklist should also equal 0.33 in the same tree.
4.	Only one taxonomic concept per checklist should map to a particular Avibase ID across all taxonomic levels. One exception is that of monotypic species and their nominal subspecies, which both refer to the same Avibase ID because they have exactly identical circumscriptions. The latter are typically not included in taxonomic checklists for that very reason. While the same exception also applies to other monotypic taxonomic levels (e.g. genera with a single species), these higher groups are generally not mapped in Avibase.
5.	Taxonomic concepts at a given taxonomic level within a checklist (e.g. species) should not map to Avibase IDs that are found along the same branch of the tree in any given arrangement. If concept A is a child of concept B, and concept B is in turn a child of concept C, only one of A, B or C should be present at the same time at the same taxonomic level.
6.	The parent-child relationships within a checklist (e.g. species and subspecies relationships) must exhibit the same parent-descendant relationships as the Avibase IDs they are mapped to. For instance, if an authority publishes the species concept *Vireo solitarius* that includes *Vireo solitarius alticola* as one of its subspecies, the Avibase ID for *Vireo solitarius alticola* also needs to be a descendant of the Avibase ID for *Vireo solitarius*.
7.	Alternate trees should share all the same terminal nodes, as well as the same top parent node, but they can have a different suite of intermediate nodes. Intermediate nodes that are shared among alternate trees should also have the same terminal children nodes in all those trees (i.e. the nodes must represent the same population of individuals in all trees).

It is possible to automate mapping of the taxonomic concepts from an authority to an Avibase ID using fractional weights and the properties of indirect dependencies. This requires examining all possible combinations of Avibase IDs that match the names present in a given authority in order to find which valid combination will provide a total fractional weight sum of 1.0 without breaking the descendant rules. For example, Avibase contains two distinct taxonomic concepts that bear the name *Vireo solitarius* (Fig. 4): *Vireo solitarius* *s.l.* (w=1.0), and *Vireo solitarius* *s.s.* (w=0.333). Taking a hypothetical example, if a comprehensive checklist from a new authority is incorporated into Avibase that only contains *Vireo solitarius*, and not the other two possible species names (*Vireo cassinii* and *Vireo plumbeus*), then this authority’s concept of *Vireo solitarius* is determined as identical to the *Vireo solitarius* *s.l.* (w=1.0) concept. If a later revision of that checklist includes all three taxa (*Vireo solitarius*, *Vireo cassinii* and *Vireo plumbeus*), Avibase could recognize that this authority’s concept matches the *Vireo solitarius* *s.s.* concept (w=0.333). If a further revision were to elevate a subspecies of *Vireo solitarius* (say, *Vireo solitarius alticola*) to a species, there would now be three separate taxonomic concepts with weights of 1.0, 0.333 and 0.167 for *Vireo solitarius* (the last matching the concept for the former nominal subspecies, which would now be treated as a full species). This process can be extended to more complicated arrangements, and as long as the relationships are properly constructed, there should always only be a single valid combination possible.

A recent attempt to use such an automated approach to mapping taxonomic concepts showed promising results. For this, we used species concepts from Peters’ *Check-List of Birds of the World*, a landmark series of books published between 1931 and 1987 and recently converted into a database (Peters et al. 1931–1987, Lepage and Warnier 2014). As with the manual mapping process, the first step was to assign scientific names to name concepts using Avibase’s extensive synonymy database. The process then looked for unique combinations of Avibase IDs that, when mapped to these particular name concepts, provided a sum of fractional weights of 1.0, and for which only one solution existed. Out of the nearly 8,900 species concepts included in Peters, the vast majority of them (97%) were successfully mapped to a unique Avibase ID on a first attempt. Upon examination, the ~300 species that could not be mapped represented either new concepts for Avibase or revealed problems with incorrectly constructed taxon trees breaking one or more of the rules listed in Table 3. With manual adjustments of the concept trees, all concepts were eventually successfully mapped to Avibase IDs.

## Mapping biodiversity data to taxonomic databases

The system of automated mapping may also be used to disambiguate taxonomic concepts in biodiversity databases. Unlike names in checklists, those attached to biodiversity data often do not contain information that reveals their circumscription, unless they were published as taxonomic concepts. Avibase has yet-untapped potential as a taxonomic concept resolution service. Users would pass a list of names to the service and Avibase would find those names across concept trees, helping to resolve any ambiguity. If these names serve as labels to other information, such as specimen records, Avibase could thus disambiguate the intended circumscription of such records. This is especially valuable for older records, which may use names and concepts long out of date.

For biodiversity databases that rely primarily on scientific names and not taxonomic concepts to index records, other properties of the records could be used to determine the intended circumscription. For instance, one could evaluate a historical record originally described as *Vireo solitarius* in Vancouver, B.C. as almost certainly referring to the current species concept *Vireo cassinii*, because the probability of observing *Vireo solitarius* *s.s.* at that location and date is minimal. A request to such a service could include some or all of the following properties for each record that needs to be assessed, represented in standard formats described in the Darwin Core standard (Wieczorek et al. 2012): scientific name, scientific name author, taxonomic concept source (authority name and publication year), vernacular names (in English or other languages), record collection site (country, state or province, county, geographic coordinates, etc.), record collection date and so on.

Such services have not yet been implemented in Avibase, providing a compelling rationale for further development, which is ongoing. Each property could be assigned a relative weight so that scores are assigned to prospective matches. Each taxonomic concept could be classified as regular, rare, or absent from a geographic area, and different scores assigned to each of these categories. This information is already available in Avibase for several thousand geographic regions (e.g. continents, countries, state/provinces, islands), and a more elaborate version could make use of increasingly available distribution data available from sources such as eBird (http://ebird.org) and Map of Life (http://mappinglife.org; Jetz et al. 2012) to assign probability scores that a particular bird taxon would be observed at a specific location and date. Different types of uses could be more tolerant to uncertainty than others, something that would be left to the discretion of the user. Fuzzy taxonomic name matching algorithms such as TaxaMatch (Rees 2009, 2011), which are particularly suited to identifying common errors in writing and transcribing taxonomic names, could also be implemented to account for variants and errors in spelling. While these methods may become increasingly refined over time, their probabilistic nature means that they will probably never fully replace the need for expert opinion. Experts and users should be able to decide how much weight is given to various considerations in the scores, and what degree of certainty is required for their specific need.

## Conclusion

Avibase provides a clear demonstration that taxonomic concepts can be successfully organized on a large scale, and perhaps more importantly, that it can be done by relying on the taxonomic “currency” already used by most practitioners, i.e. the names published in the form of authoritative checklists. We hope and expect that other researchers will continue to further develop the ontological framework and the database models that are needed to organize concepts, populate databases and build relationships, as well as develop services that will allow interacting with these systems. As global biodiversity databases aggregated from various sources continue to grow in size and in scope, and as taxonomic advances continue, the deficiencies of relying solely on scientific names should become increasingly apparent. The use of taxonomic concepts in place of scientific names, together with the development of taxonomic concept databases and easily available resolution services, would be a major step forward in addressing some of these issues, and in facilitating the paradigm shift needed to transition from taxonomic names to taxonomic concepts.

## Dealing with uncertainty

Whether or not automation is used, there will be instances where circumscriptions cannot be established with full confidence, particularly when checklist authorities provide incomplete information. An example is the African parrot subspecies *suahelicus*, found from Tanzania to Angola and northeastern South Africa, which has been alternatively included as part of *Poicephalus robustus* or *Poicephalus fuscicollis* by various authors, thus creating two possible distinct species concepts for each name, each with very different definitions. In one case, the nominate *Poicephalus robustus robustus* endemic to South Africa is combined with *Poicephalus robustus suahelicus*, and the monotypic species *Poicephalus fuscicollis* is restricted to western Africa from Gambia to Angola. In the alternative treatment, the monotypic species *Poicephalus robustus* is restricted to South Africa, and the subspecies *suahelicus* is combined with *Poicephalus fuscicollis*, covering most of sub-Saharan Africa. In a checklist or publication that contains only the two species names *Poicephalus robustus* and *Poicephalus fuscicollis*, without a list of subspecies or phenotypic characteristics (such as range or plumage) which might disambiguate the possible circumscriptions, there will be uncertainty in mapping those concepts to the correct Avibase IDs. In such cases, Avibase relies on circumstantial evidence to help with the mapping process. If, for instance, one of the two arrangements had not been recognized as valid for quite some time, it may be safe to assume that the authors intended to refer to the contemporary concepts. A more refined approach would be to categorize the criteria used to establish each mapping and the degree of uncertainty attached to it, something that Avibase has not yet dealt with.

Another approach to dealing with those uncertain cases could be to create new Avibase IDs for each of those poorly defined nominal concepts, with their own partially constructed relationship tree (e.g. without subspecies nodes). Because the taxonomic tree of relationships in those cases is incomplete, there will be uncertainty in establishing the relationship of those poorly defined taxonomic concepts to other concepts in alternate arrangements. The framework proposed by Franz and Peet (2009) allows describing these types of relationships that involve poorly defined concepts by combining expressions with the symbol OR. For instance, if two taxonomic concepts share the same name, they can be assumed to at least partly overlap and may possibly also be entirely congruent, something that can be captured in the table of Other Relationships in the Avibase database model (Fig. 1). While this is a problem that Avibase has not yet attempted to address, this issue will be mainly limited to nominal concepts, published without sufficient information to allow proper mapping, which does not usually apply to authoritative checklists mapped into Avibase.
